# Factors impacting on diabetes knowledge, medication adherence and glycemic control among adult diabetics visiting a county teaching and referral hospital in Kenya: a cross-sectional study

**DOI:** 10.11604/pamj.2021.40.5.21848

**Published:** 2021-09-02

**Authors:** Faith Machocho Mwangasha, David Gitonga Nyamu, Lucy Jemutai Tirop

**Affiliations:** 1 Coast Teaching and Referral Hospital, P.O. Box 90231-80100, Mombasa, Kenya,; 2 School of Pharmacy, University of Nairobi, P.O. Box 19676-00202, Nairobi, Kenya

**Keywords:** Diabetes knowledge, medication adherence, glycated haemoglobin, glycemic control

## Abstract

**Introduction:**

optimal management of diabetes involves interplay between patients’ understanding of their disease and medication adherence, which would eventually influence glycemic control. However, there is scant published literature on the interconnection of patient related factors that impact on optimal management of diabetes in resource-limited settings such as Kenya.

**Methods:**

a hospital based cross-sectional survey involving 270 freely consenting adult diabetics investigated the interconnection between diabetes knowledge, medication adherence and glycemic control. Data on the patient´s knowledge and medication adherence was collected using validated tools whilst glycemic control was evaluated using the patients' glycated haemoglobin values. Data analysis was carried out using STATA version 13 statistical software, employing the chi square test for association and simple linear regression for prediction, with p ≤0.05 considered significant.

**Results:**

participants´ level of knowledge on diabetes was significantly associated with the academic achievement (p=0.001), while their medication adherence was significantly associated with family support (p=0.001) and duration of disease since diagnosis (p=0.019). On linear regression, family support occasioned by the nature of participants’ household setup, had a strong positive correlation [r=0.99 (CI 0.60-1.00)] with optimal glycemic control.

**Conclusion:**

family support is an important determinant of medication adherence and optimal glycemic control among diabetic patients. Clinicians should team up with family members of diabetic patients for optimal glycemic control.

## Introduction

**Scientific background:** the optimal management of chronic illnesses such as diabetes is influenced by environmental, patient-related, health system and disease-related factors [1]. As the patient is the key determinant of the success of treatment, patient-related factors, though complex, form the backbone of optimal diabetes control [2]. Among these patient related factors are diabetes knowledge, medication adherence, economic and psychosocial factors.

Patient knowledge comprises the extent of the patients´ understanding of diabetes as to what the disease is, its complications and both its pharmacological and non-pharmacological management [3]. Patient knowledge has been shown to be dependent on socio-demographic factors such as age, sex and literacy level [3], the ability to access information as well as the accuracy and presentation of medical information [4].

Medication adherence entails the willingness and the ability of the patient to comply with the prescribed treatment regimen [5]. Non-adherence may be classified into two levels: intentional and non-intentional. Intentional non-adherence originates from the patients´ choice not to adhere to a drug regimen while non-intentional non-adherence is occasioned by factors beyond the patient´s control such as unavailability of drugs from the local pharmacy [6]. Non-adherence to medication also includes a deliberate choice on the patients´ part not to even start on a treatment regimen [5].

**Rationale:** patient related factors, which are the most important determinants of the success of diabetes treatment, have not been adequately evaluated in a low resource setting such as Kenya.

**Study objectives:** the present study determined and explored the interplay between patient knowledge, medication adherence and glycemic control among adult diabetics in a low resource setting.

## Methods

The study design was a cross-sectional survey conducted at the Diabetes Outpatient Clinic in Coast Teaching and Referral Hospital (CTRH) in Mombasa, Kenya. The hospital is the largest teaching and referral public hospital in Mombasa County with a bed capacity of 1000. The study population included adult (age ≥ 18 years) patients, attending the clinic at hospital, who had been medication for over two months. Both sexes were included in the study. Pregnant women, patients with mental illness or liver disorders were excluded from the study.

Ethical approval for the study was obtained from both the Kenyatta National Hospital-University of Nairobi Ethics Review Committee (KNH-ERC/A/259) and the CTRH Ethics Review Committee (ERC-CGH/MSc/VOL.1/62). Data on glycated haemoglobin (HbA1c), which correlated with glycemic control, was extracted from patient files, whilst that on socio-demographics and patient-related factors was collected using pre-designed study questionnaires. The target sample size was 270 patients and was estimated via the Cochrane formula applying a desired precision of 0.05 and medication adherence level of 0.5 [7]. Convenient sampling was used until the sample size was achieved.

**Missing data:** where HbA1c data was unavailable, fasting blood sugar and post prandial blood sugar values were used to evaluate glycemic control.

**Bias:** confounding factors such as co-morbidities and intrinsic genetic factors which may influence glycemic control were not accounted for in the study.

*Patient Knowledge* was investigated using a revised and validated Diabetes Knowledge Test (DKT) adopted from Michigan Diabetes Research Center [8]. The DKT comprise of twenty statements about diabetes to which the participant could respond true (1 mark), false (1 mark) or don´t know (0 mark). The respondent´s final score was expressed out of 20 then computed as a percentage.

*Medication Adherence levels* were assessed by the use of the Morisky Medication Adherence Scale (MMAS), adopted from the International Journal of Clinical and Health Psychology [9]. The respondent´s level of adherence was measured by the use of a series of eight questions, each with structured responses resembling the Likert Scale of 1-5 attached to a score. The score was totaled out of 40 and expressed as a percentage. The higher scoring patient was deemed to be more adherent than the lower scoring patient. The nature of non-adherence, whether intentional or non-intentional, was determined by the nature of responses given.

The DKT and MMAS scores were then correlated with the presumed determinants of patient knowledge (participants´ age, sex, economic capacity and education level) and medication adherence (monthly income, disease duration, household set up) using the Chi square test for association. The determinants shown to have a significant association with knowledge levels or medication adherence among the participants were then correlated to glycemic control using simple linear regression.

## Results

**Socio-demographic characteristics of the participants:** the socio-demographic characteristics of 270 patients who participated in the study are presented in Table 1. The study participants had a slight male preponderance (53.7%) with the majority being middle-aged adults of 41-50 years (29.3%) and 31-40years (19.3%). The middle-income bracket, (KES 20001-30000), typified 32.2% of the participants as the modal income. Most of the participants were of either secondary (46.2%) or tertiary education levels (43.3%). The majority of the participants were married (39.2%) and lived with their families (64.4%) (Table 1).

**Table 1:** socio-demographic characteristics of the participants

Variable	Category	n	Percentage (%)
**Gender**	**Male**	**150**	**53.7**
	Female	120	46.3
**Age(Years)**	20-30	25	9.2
	31-40	52	19.3
	**41-50**	**79**	**29.3**
	51-60	64	23.7
	+60	50	18.5
**Marital Status**	Single	38	14.1
	Cohabiting	26	9.6
	**Married**	**106**	**39.2**
	Separated	39	14.4
	Divorced	21	8.1
	Widowed	38	14.1
	Missing	2	0.5
**Medical Insurance**	Yes	139	51.5
	Partial	21	7.8
	**No**	**110**	**40.7**
**Monthly Income (Kenya Shillings)**	≤10000	21	7.8
	10001-20000	32	11.8
	**20001-30000**	**87**	**32.2**
	30001-40000	52	19.3
	40001-50000	43	15.9
	+50000	35	13.0
**Employment Status**	**Formally Employed**	**103**	**38.1**
	Self-employed	96	35.5
	Unemployed	34	12.6
	Pensioned	30	11.2
	Job-seeking	7	2.6
**Religious Affiliation**	**Staunch**	**77**	**28.5**
	Moderate	91	33.7
	Basic	77	28.5
	Other	22	8.5
	Missing	3	1.1
**Highest Education level**	Primary	17	6.7
	**Secondary**	**125**	**46.2**
	Tertiary	117	43.3
	Informal	9	3.3
	Missing	2	0.4
**Living Arrangement**	**With Family**	**174**	**64.4**
	With Partner	27	10
	With Friends	3	1.1
	With Colleagues	4	1.5
	Alone	62	23

**Clinical characteristics of the participants:** most of the participants (58.9%) reported 6-10 years of disease duration (Table 2). Diabetes support group participation was low among the study population, at only 21.5%. Optimal glycemic control (HbA1c <7%) was reported among 67.8% of the participants (Table 2).

**Table 2:** clinical characteristics of the participants

Variable	Category	N	Percentage (%)
**Disease duration, years**	≤ 5	61	22.6
	**6-10**	**159**	**58.9**
	11-15	47	17.4
	16+	1	0.4
	Missing	2	0.7
**In DM Support Group**	Yes	58	21.5
	**No**	**212**	**78.5**
**Optimal Glycemic Control**	**Yes**	**183**	**67.8^†^**
	No	84	31.1
	Missing	3	1.1
**HbA1c Range (%)**	6-6.5	60	22.2
	**>6.5-7**	**80**	**29.6**
	>7-8	20	7.4
	>8-9	51	18.8
	>9	13	4.8
	Other*	43	15.9
	Missing	3	1.1

Key: DM=Diabetes Mellitus and HbA1c=Glycosylated haemoglobin*Where HbA1c values were unavailable Fasting Blood Sugar and Post Prandial Blood Sugar values were used to establish glycemic control. ^†^43 patients (15.9%) were deemed to have optimal glycemic control via Fasting Blood Sugar and Post Prandial Blood Sugar values.

**DKT score:** the average score of the participants in the DKT was 42.7%. Most of the participants had an average knowledge level of diabetes, with 44.8% of them scoring between 31-50% in the test. Almost a third (29.6%) of the participants had DKT scores of 51-75%, representing an above average knowledge level. A small proportion of 6.6% and 18.5% of the participants had DKT scores of > 75% (excellent level of knowledge) and ≤ 30% (low level of knowledge), respectively. Among participants perceived to have excellent diabetes knowledge (DKT score > 75%), majority had attained tertiary level of education at 88.9% (Table 3). Conversely, the majority of participants scoring the lowest on the DKT (≤ 30%) had attained primary or secondary education. Table 4 elucidates some DKT responses by this lowest scoring group.

**Table 3:** DKT score vs gender and education level of respondents

Variable	Category	n	Percentage (%)
**DKT ≤30**	Gender		
	**Male**	**27**	**54**
	Female	23	46
	Highest Education Level		
	Informal	9	18
	Primary	17	34
	**Secondary**	**22**	**44**
	Tertiary	2	4
**DKT +75**	Gender		
	**Male**	**10**	**55.6**
	Female	8	44.4
	Highest Education Level		
	Informal	0	0
	Primary	0	0
	Secondary	2	11.1
	**Tertiary**	**16**	**88.9**

**Table 4:** most poorly done questions on diabetes knowledge test

Variable	Category	n	Percentage
**Glycosylated hemoglobin (HbA1c) is a test that measures your average blood glucose level in the past week**.	**Correct**	**5**	**10**
	Wrong	30	60
	Don’t know	15	15
**Urine testing and blood testing are both equally as good for testing blood glucose**.	**Correct**	**3**	**6**
	Wrong	40	80
	Don’t know	7	14
**Wearing shoes a size bigger helps prevent foot ulcers**.	**Correct**	**2**	**4**
	Wrong	45	90
	Don’t know	3	6
**High blood glucose may be caused by too much insulin**.	**Correct**	**1**	**2**
	Wrong	46	92
	Don’t know	3	6
**If you take your morning insulin and skip breakfast your blood glucose levels normally decrease**.	**Correct**	**2**	**4**
	Wrong	45	90
	Don’t know	3	6
**Attending your doctor’s appointments will stop from you getting diabetes complications**.	**Correct**	**1**	**2**
	Wrong	48	96
	Don’t know	1	2

The number of correct responses for each variable is highlighted.

**MMAS score:** slightly over half (56.6%) of the study participants recorded an above average level of medication adherence (MMAS-8 scores above 50%). Lower levels of adherence, MMAS-8 scores of 31-50% and ≤ 30% were occasioned by 31.5% and 11.9% of the participants, respectively. Table 5 profiles the treatment adherence patterns with possible determinants namely participant income, disease duration and living arrangement.

**Table 5:** treatment adherence patterns of the participants relative to income, disease duration and living arrangement

Variable	Category	N	Percentage (%)
**MMAS-8 score ≤30 (Least Adherent)**	Monthly Income (KES)		
	**≤10000**	**20**	**62.5**
	10001-20000	3	9.4
	20001-30000	1	3.1
	30001-40000	1	3.1
	40001-50000	1	3.1
	+50000	6	18.8
	Disease Duration (Years)		
	**≤ 5**	**20**	**62.5**
	6-10	3	9.4
	11-15	9	28.1
	Living Arrangement		
	With Family	4	12.5
	Partner	10	31.3
	**Alone**	**18**	**56.2**
**MMAS-8 score +75 (Most Adherent)**	Monthly Income (KES)		
	≤10000	0	0.0
	10001-20000	2	6.25
	20001-30000	3	9.4
	30001-40000	5	15.6
	40001-50000	2	6.25
	**+50000**	**20**	**62.5**
	Disease Duration (Years)		
	≤ 5	5	15.6
	6-10	5	15.6
	**11-15**	**22**	**68.8**
	Living Arrangement		
	**With Family**	**15**	**46.9**
	Partner	10	31.2
	Alone	7	21.9

Key: MMAS-8=Morisky Medication Adherence Scale version 8, KES= Kenya Shillings the highest values are highlighted in each category.

**Associations between the predictor factors and the first outcome variables:** the associations between the participants´ socio-demographic characteristics and diabetes knowledge and treatment adherence were determined using the chi square test. There was a statistically significant association between the participants´ knowledge level and their literacy level (p=0.001). The effect of participants´ age (p=0.533) and disease duration (p=0.605) on their knowledge levels were non-significant. On the contrary, disease duration (p=0.019) and household setup (p=0.001) were significantly associated with treatment adherence. The relationship between participants´ monthly income and treatment adherence was not substantial (p=0.206).

**Correlation between the patient socio-demographics and clinical characteristics with optimal glycemic control:** the correlation between the predictors associated with the aforementioned patient factors and optimal glycemic control was computed using simple linear regression. The education level of the participants showed only a weak positive correlation [r=0.23 (CI -0.94-0.98)] with optimal glycemic control. The disease duration had a weak negative correlation [r=-0.22 (CI -0.97-0.94)] to optimal glycemic control. Notably, the nature of household setup posted a strong positive correlation [r=0.99 (CI 0.60-1.00)] to optimal glycemic control among study participants.

## Discussion

The number of participants with optimal glycemic control was higher than that observed in similar studies; for instance a study done in Kenyatta National Hospital, the largest referral hospital in Kenya, where optimal glycemic control was recorded by 36.9% of the sample population [7]. This observation may be indicative of improvement in patient management. Among the fifty participants recording the lowest DKT scores (≤ 30%), questions on diabetes complications and insulin therapy were the most poorly done. Notably, the deficiency of patients´ knowledge on insulin therapy has been reported. A recent study by Kituzi *et al*. [10] revealed that only 53.4% of adult diabetics visiting a Kenyan referral hospital correctly performed the all steps of the insulin injection technique as described by the American Diabetes Association.

The participants´ level of income and disease duration were positively correlated with medication adherence. High-income earners (KES+50000) and those with longer disease duration (11-15 years) dominated the MMAS-8 score > 75% bracket. Participants living with their families recorded higher adherence scores than those living alone. These findings are consistent with those reported from related studies [7, 11, 12]. Probably higher income may enhance affordability of medication [7], whilst patients with longer duration of disease are more likely to interact better with their health care providers, understand their treatment plans, and become more aware of their diseases [12]. Patients were shown to value family support, which perhaps involved reminders of medication times, preparation of healthy meal plans and participation in exercise sessions, towards medication adherence often with positive results [11]. Among the study participants attaining the lowest MMAS-8 scores (≤ 30%), 55% expressed non-adherence occasioned by factors such as forgetfulness, implicative of non-intentional non-adherence.

The weak correlation of participants´ education level with optimal glycemic control, implied that whilst patient knowledge is essential, there may be other intervening factors affecting actual glycemic control. Factors that promote self-monitoring, diet and exercise adherence must also be taken into account [13]. The weak negative correlation of disease duration to optimal glycemic control has been reported previously [14]. The nature of household setup was a strong determinant of glycemic control, with those participants living with their families recording lower HbA1c values. As stated earlier, patients greatly valued family support towards medication adherence and overall glycemic control [15].

**Limitations:** while we appreciate that the strength of the present study is use of validated data collection tools, a potential limitation is the inclusion of patients primarily from a referral hospital in the coastal region of Kenya as a representation of the general Kenyan population. Additionally, as common with cross-sectional studies involving direct interviews, patients could have understated or overstated their experiences.

**Generalisability:** the generalisability of the study findings elsewhere in Kenya warrants further investigation. Notably, the study population has unique cultural practices and norms compared to the rest of the country.

## Conclusion

Patients´ literacy level, a determinant for knowledge showed a weak positive correlation to optimal glycemic control among the study participants. Living within a family setup was significantly associated with better treatment adherence and positively correlated to optimal glycemic control. Involvement of family members in the diabetic patients´ hospital visits and treatment plans should be encouraged.

### What is known about this topic

- Successful management of diabetes mellitus is influenced by a multitude of factors; environmental, health system, patient-related and disease-related factors;
- Patient related factors form the backbone for optimal management of diabetes mellitus;
- Among these patient related factors are diabetes knowledge, medication adherence, economic and psychosocial factors.

### What this study adds

- The current study elucidates the interplay of factors between patient knowledge, medication adherence and glycemic control among adult diabetics in a low resource setting;
- Education level, a determinant for patient knowledge, showed a weak positive correlation to optimal glycemic control;
- Family support, a determinant for medication adherence, showed a strong positive correlation to optimal glycemic control.
